# CFTR mutations and genotypic patterns among cystic fibrosis patients with allergic bronchopulmonary aspergillosis: analysis from a tertiary care center in Saudi Arabia

**DOI:** 10.3389/fped.2026.1938948

**Published:** 2026-08-27

**Authors:** Maryam Fuad Ali, Hessa AlOtaibi, Talal AlZahrani, Bushra Hafez, Ayesha Khokhar, Maad Jalal, Amal Albalawi, Raghad AlHuthil, Hanaa Banjar

**Affiliations:** 1 Department of Pediatrics, Arabian Gulf University, Manama, Bahrain; 2 Department of Pediatrics, King Fahad Specialist Hospital, Dammam, Saudi Arabia; 3 Department of Pediatrics, King Faisal Hospital and Research Centre, Medina, Saudi Arabia; 4 Al Faisal University, Riyadh, Saudi Arabia; 5 Department of Pediatrics, Hamad Medical Corporation, Doha, Qatar; 6 Yale Biomedical Imaging Institute, New Haven, CT, United States; 7 Department of Pediatrics, King Faisal Hospital and Research Centre, Riyadh, Saudi Arabia

**Keywords:** allergic bronchopulmonary aspergillosis, *Aspergillus*, CFTR, cystic fibrosis, genotype

## Abstract

**Introduction:**

Cystic fibrosis (CF) is a multisystem disease caused by mutations in the cystic fibrosis transmembrane conductance regulator (CFTR) gene. Allergic bronchopulmonary aspergillosis (ABPA) is a recognized complication in CF, with genetic variability potentially influencing susceptibility. This study aims to assess the distribution of CFTR genotype classes in CF patients with ABPA and explore their association with disease severity and baseline pulmonary function.

**Methods:**

A retrospective cross-sectional study was conducted over a 10-year period, including patients <18 years with genetically confirmed CF and concomitant ABPA. CFTR mutation data were analyzed, and comparative analyses were performed across CFTR genotype classes.

**Results:**

Twenty-two patients were included, with a median age of 13.75 years at the time of ABPA diagnosis. CFTR mutations identified were predominantly classified as Class I (72.7%), followed by Class II (22.7%), with a single patient harboring a Class V mutation. Patients with Class II/V mutations showed lower growth indices and baseline pulmonary function compared to Class I, without statistical significance (*p* > 0.05). Chest CT findings were comparable between the groups, with bronchiectasis and bronchial wall thickening representing the most common radiological abnormalities.

**Conclusion:**

CFTR genotype may influence clinical phenotype in CF patients with ABPA, with trends toward poorer growth and lung function in more severe mutation classes, although larger studies are needed to confirm these associations.

## Introduction

Cystic fibrosis (CF) is an inherited autosomal recessive disorder affecting multiple organ systems, resulting from mutations in the cystic fibrosis transmembrane conductance regulator (CFTR) gene (1). Previous studies have demonstrated an increased predisposition to atopic conditions among individuals with CF, among which allergic bronchopulmonary aspergillosis (ABPA) is particularly significant (2). ABPA is an immune-mediated hypersensitivity reaction triggered by airway colonization with *Aspergillus* species, most commonly *Aspergillus fumigatus*. It is estimated to affect a considerable proportion of pediatric patients with CF, with prevalence rates approaching 10% (3, 4). In the absence of timely recognition and appropriate management, ABPA may lead to recurrent pulmonary exacerbations, progressive structural lung damage, and sustained decline in respiratory function (4).

Identifying risk factors for the development of ABPA in patients with CF is of critical importance, as it may facilitate earlier surveillance, timely diagnosis, and prompt initiation of appropriate therapy. Among these, the potential association between ABPA and specific CFTR genotypes has gained increasing attention, as genetic variability may influence susceptibility to this complication (5).

The distribution of CFTR genotypes among a large cohort of over 300 patients with CF in Saudi Arabia has been previously characterized in a recent retrospective study, demonstrating substantial allelic heterogeneity and predominance of homozygous variants, likely reflecting the high rates of consanguinity in the region (6).

In the present study, we aim to investigate the distribution of CFTR genotypes among patients with CF diagnosed with ABPA and to examine potential associations between specific genotype classes and clinical characteristics, including disease severity and baseline pulmonary function.

## Materials and methods

This retrospective cohort study included pediatric patients with CF who were followed at the Pediatric Cystic Fibrosis Clinic of a tertiary care center in Riyadh, Saudi Arabia. Patients were identified through a review of institutional electronic medical records over a 10-year period; from January 2015 to January 2025. Eligibility criteria included a genetically confirmed diagnosis of CF via the demonstration of two pathogenic CFTR variants either in homozygous or compound heterozygous state, age younger than 18 years at the time of ABPA diagnosis, and fulfillment of the International Society for Human and Animal Mycology (ISHAM) diagnostic criteria for ABPA (7).

A total of 24 eligible patients were identified. Two patients were excluded because of insufficient clinical documentation, resulting in a final study cohort of 22 patients.

Demographic and clinical data were retrospectively extracted from the electronic medical record system using a standardized data collection form. Variables collected included age at ABPA diagnosis, sex, region of origin within Saudi Arabia, and CFTR genotype. Identified CFTR variants were categorized according to their established functional mutation classes (Classes I-V). Patients were subsequently stratified by mutation class to evaluate genotype-phenotype associations through comparative analyses.

Chest CT findings were extracted from consultant radiologist reports available in the electronic medical records. Spirometry was performed in accordance with the American Thoracic Society (ATS) guidelines using calibrated equipment. Pulmonary function parameters were interpreted using age-, sex- and height-specific reference values (8).

### Data analysis

Data analysis was done using STATA version 18. Continuous variables were reported as medians and interquartile ranges (IQR) because of the small sample size and non-normal distribution, while categorical variables were presented as frequencies and percentages. Baseline growth and lung function by CFTR mutation class were assessed using the Mann–Whitney *U* test, a non-parametric test appropriate for continuous data from two independent groups. Baseline chest CT was assessed using Fisher's exact test because of the small sample size. A *P* value of <0.05 was considered statistically significant.

### Ethical approval

This study was conducted in accordance with the ethical principles outlined in the Declaration of Helsinki. Ethical approval was obtained from the Research Advisory Committee (RAC) at King Faisal Hospital and Research Center (KFSHRC), Riyadh, Saudi Arabia (RAC number: 2221214) prior to commencement of data analysis. Due to the retrospective nature of the study and the use of anonymized data, informed consent was not applicable to the study design.

## Results

Baseline demographic and clinical characteristics of the study population are summarized in Table 1. The cohort consisted of 22 pediatric patients with CF diagnosed with ABPA. The median age at diagnosis was 13.75 years (IQR: 12.1–17.1 years). Females accounted for 54.6% of the cohort (*n* = 12), while males represented 45.5% (*n* = 10). All patients were of Saudi nationality. Parental consanguinity was reported in 17 patients (77.3%).

**Table 1:** Demographics and clinical data of 22 CF patients with ABPA.

Variables	*n* (%), Median (IQR)
Sex	
Female	12 (54.6)
Male	10 (45.5)
Saudi Region	
Central	5 (22.7)
Eastern	7 (31.8)
Northern	3 (13.6)
Southern	7 (31.8)
Consanguinity	
Yes	17 (77.3)
No	5 (22.7)
Age at diagnosis (years)	13.75 (12.1–17.1)
Sweat Test Value (*n* = 20)	98.5 (83.5–102.5)
Genetic testing	
CFTR positive	22 (100)
CFTR mutation class (*n* = 22)	
Class I – Defective protein production (no synthesis)	16 (72.7)
Class II – Defective protein processing/trafficking	5 (22.7)
Class V – Reduced protein synthesis (quantity)	1 (4.6)

IQR, interquartile range; CFTR, cystic fibrosis transmembrane conductance regulator.

Sweat chloride testing was performed in 20 patients, all of whom demonstrated elevated levels, with a median value of 98.5 mmol/L (IQR: 83.5–102.5).

The majority of patients, 14 (63.6%), were chronically colonized with mucoid *Pseudomonas aeruginosa*, including one patient with concomitant *Candida* spp. colonization. Methicillin-sensitive *Staphylococcus aureus* and methicillin-resistant *Staphylococcus aureus* were isolated in three (13.6%) and two (9.1%) patients, respectively. Single cases of *Stenotrophomonas maltophilia* and *Mycobacterium avium* complex colonization were identified. One patient had no positive respiratory cultures.

All patients received omalizumab for the treatment of ABPA. Concomitant antifungal therapy and systemic corticosteroids were administered to 19 (86.4%) and 11 (50.0%) patients, respectively, while none were receiving CFTR modulator therapy.

Genetic testing for CFTR mutations was positive in all patients (100%). Among those with identified mutations, Class I variants were the most common, accounting for 72.7% of cases, followed by Class II mutations in 22.7%, and one patient with a Class V mutation. The specific CFTR variants identified in the cohort are detailed in Table 2.

**Table 2:** CFTR gene variants identified in 22 CF patients with ABPA.

Region: Variant	Zygosity	Coding impact	ACMG class	CFTR class	dbSNP	*n*
intron5:c.579+1G>A	Homozygous	Non-coding	P	class I	rs77188391	4
intron5:c.579+1G>A & exon13:c.1703T>A; p.(Leu568Ter)	Compound heterozygous	Non-coding & Nonsense	P	class I	rs77188391, rs397508273	1
exon10:c.1375_1383del; p.(Ser459_Gly461del)	Homozygous	In-frame	LP	class II	rs786205658	1
exon11:c.1399C>T; p.(Leu467Phe)	Homozygous	Missense	LP	class II	rs1800089	1
exon11:c.1418del; p.(Gly473GlufsTer54)	Homozygous	Frameshift	P	class I	rs397508205	4
exon11:c.1522_1524del; p.(Phe508del)	Homozygous	In-frame	P	class II	rs121909001	3
exon11:c.1549del; p.(Tyr517ThrfsTer10)	Homozygous	Frameshift	LP	class I	NA	1
exon14:c.1911del; p.(Gln637HisfsTer26)	Homozygous	Frameshift	P	class I	rs1554389296	1
intron18:c.2988+1G>A	Homozygous	Non-coding	P	class I	rs75096551	2
intron18:c.2988+1G>A & exon20:c.3294G>A; p.(Trp1098Ter)	Compound heterozygous	Non-coding & Nonsense	P	class I	rs75096551, rs397508533	1
delExon19–21	Homozygous	Frameshift	P	class I	NA	2
exon22:c.3700A>G; p.(Ile1234Val)	Homozygous	Missense	P	class V	rs75389940	1

CFTR, cystic fibrosis transmembrane conductance regulator; P, pathogenic; LP, likely pathogenic; NA, not available; dbSNP, single nucleotide polymorphism database.

A comparative analysis was conducted between patients harboring Class I CFTR mutations and those with Class II or Class V mutations. Individuals with Class II and V mutations showed lower median height *z*-scores and reduced baseline pulmonary function compared with patients with Class I mutations. Nevertheless, these observed differences were not statistically significant (*p* > 0.05).

Baseline chest CT findings were similar between patients with CFTR Class I and Class II or Class V mutations, with high prevalence of bronchiectasis and bronchial wall thickening in both groups. No statistically significant differences were observed (*p* > 0.05). The results of the comparative analysis between CFTR mutation classes are summarized in Tables 3, 4.

**Table 3:** Baseline growth parameters and lung function by CFTR mutation class.

Measure	Class I (*n* = 16) Median (IQR)	Class II or V (*n* = 6) Median (IQR)	*P*-value
Growth Parameters			
Height-for-age *z*-score	−1.13 (−2.16 to −0.38)	−2.18 (−2.27 to −1.23)	0.268
Weight-for-age *z*-score	−1.69 (−3.15 to −0.78)	−1.85 (−2.88 to 2.04)	0.825
Pulmonary Function Tests			
Actual FVC (L)	1.15 (0.94–1.36)	1.10 (0.80–1.34)	0.805
Predicted FVC (%)	66 (45–81)	54.5 (38–70)	0.302
Actual FEV1 (L)	0.95 (0.76–1.18)	0.88 (0.59–1.11)	0.564
Predicted FEV1 (%)	68 (49–77)	50 (30–63)	0.161
FEV1/FVC Ratio (%)	86 (77–93)	82 (78–83)	0.265
Actual FEF25–75 (L)	1.18 (0.79–1.82)	0.76 (0.66–1.27)	0.248
Predicted FEF25–75 (%)	63.5 (47–98)	32 (25–57)	0.108

FVC, forced vital capacity; FEV1, forced expiratory volume in one second; FEF25–75, forced expiratory flow at 25%–75% of pulmonary volume; IQR, interquartile range.

*P*-values from the Mann–Whitney *U* test.

**Table 4:** Baseline chest computed tomography scan findings by CFTR mutation class.

Radiological finding	Class I (*n* = 13)	Class II or more (*n* = 4)	*P*-value
Diffuse Infiltrate	6 (46.2)	2 (50.0)	1.000
Bronchiectasis	11 (84.6)	4 (100.0)	1.000
Bronchial Wall Thickening	10 (76.9)	3 (75.0)	1.000
Nodular Changes	1 (7.7)	0 (0.0)	NA
Cystic Changes	2 (15.4)	1 (25.0)	1.000
Interstitial Changes	2 (15.4)	0 (0.0)	NA
Atelectasis Changes	2 (15.4)	1 (25.0)	1.000
Increased Vascularity	3 (23.1)	0 (0.0)	NA

*P*-values were reported using Fisher's exact test.

## Discussion

Efforts to elucidate the relationship between ABPA in patients with CF and specific CFTR genotypes have been ongoing for several decades. These investigations have aimed to facilitate earlier identification of at-risk individuals, enabling timely diagnosis, earlier initiation of appropriate therapy, and ultimately improved clinical outcomes (9).

The spectrum of CFTR variants identified in our cohort exhibited partial concordance with those previously characterized locally among CF patients in Saudi Arabia. In the investigation conducted by Banjar et al., the c.1418delG mutation emerged as the predominant allele, accompanied by several recurrent variants including c.3700A>G, p.Phe508del, c.579+1G>T, and c.2988+1G>A (6). While these mutations were likewise detected in our study cohort, their relative distributions diverged. Notably, the c.1418delG mutation, recognized as the most prevalent allele in the broader CF population, was observed at a comparatively reduced frequency among patients diagnosed with ABPA. Conversely, splice-site variants such as c.579+1G>A and c.2988+1G>A alongside other class I mutations, were proportionally enriched. These data indicate that the genotypic architecture of CFTR among individuals with CF complicated by ABPA may differ from that observed in the local CF population. Nevertheless, the high prevalence of parental consanguinity observed in our cohort (77.3%) was comparable to the 85% consanguinity rate reported by Banjar et al. in their genotype study of Saudi patients with CF.

In an observational study by Chesshyre et al. evaluating patients with CF and ABPA within a comparable age group, the p.Phe508del mutation was identified as the most prevalent genotype, occurring in 56.8% of the study population (10). Similarly, Noni et al., in a cohort of Greek pediatric patients with CF-associated ABPA, reported that p.Phe508del was the predominant CFTR variant (60.3%), followed by a Class I splice-site variant; c.489+1G>T (10.3%) (11). These findings likely reflect the underlying distribution of CFTR mutations in Caucasian populations, where p.Phe508del represents the most common variant (12). In contrast, CFTR mutation patterns in Saudi Arabia differ substantially, which may account for the lower frequency of this mutation observed in our cohort. In an earlier epidemiological study, Mastella et al. did not identify a significant association between ABPA and specific CFTR genotypes. The prevalence of ABPA was found to be comparable across individuals carrying the most common CFTR mutations in European populations, suggesting that genotype alone may not be a primary determinant of susceptibility to ABPA (13). Likewise, in a recent large French registry-based case-control study, Tarizzo et al. found no significant association between CFTR genotype and the development of ABPA in people with CF, either in univariate or multivariate analyses (14).

In our cohort, Class I CFTR mutations were the most frequently identified. Similarly, a systematic review investigating the relationship between ABPA and CFTR mutations reported a predominance of Class II variants, which are also associated with severe impairment of CFTR function (5). Taken together, these observations raise the possibility of a relationship between severe CFTR dysfunction and susceptibility to ABPA. However, given the relatively small sample size of our study, the predominance of Class I mutations should be primarily considered descriptive of the CFTR genotype distribution among Saudi patients with CF-associated ABPA. Whether severe CFTR dysfunction influences susceptibility to ABPA requires further investigation in larger controlled studies.

In our cohort, patients with Class II or V CFTR mutations and ABPA demonstrated lower baseline lung function and reduced growth *z*-scores than those with Class I mutations; however, these differences did not reach statistical significance. Direct comparison with the existing literature is limited, as previous studies evaluated differences between CF patients with and without ABPA rather than across CFTR mutation classes within patients with ABPA. For example, Chesshyre et al. reported lower baseline lung function, yet higher body mass index, in CF patients with ABPA compared with those without ABPA (10). Similarly, Mastella et al. observed a 10% lower forced expiratory volume in one second (FEV1) among CF patients with ABPA compared with those without ABPA (13).

Investigating the relationship between CFTR mutations and ABPA has become increasingly important in the era of CFTR modulator therapies. Emerging evidence from epidemiological and retrospective studies suggests a reduction in the incidence and severity of ABPA among patients receiving CFTR modulators (1). These observations highlight the potential influence of CFTR function on susceptibility to ABPA and underscore the clinical relevance of genotype–phenotype associations. Accordingly, a better understanding of how specific CFTR mutations relate to the risk of ABPA may help inform risk stratification, optimize therapeutic decision-making, and further clarify the mechanisms underlying this complication.

This study is limited by its retrospective design and small sample size, which may hinder the generalizability of the findings. Larger, well-designed studies are therefore needed to better define these associations and validate current findings. Nevertheless, it provides valuable data on the CFTR mutation spectrum among Saudi pediatric patients with CF-associated ABPA, an underrepresented population.

## Conclusion

In this retrospective study of pediatric patients with CF-associated ABPA from Saudi Arabia, Class I CFTR mutations were the predominant genotype. Although in our cohort, patients with Class II and V mutations had poorer baseline growth and pulmonary function, no statistically significant genotype-phenotype associations were identified. These findings contribute to the limited regional data on CFTR genotypes in CF-associated ABPA and thereafter, highlight the need for larger, multicenter studies to better define the role of genotype in disease susceptibility and outcomes, particularly in the evolving era of CFTR modulator therapy.

## Data Availability

The raw data supporting the conclusions of this article will be made available by the authors, without undue reservation.
